# The IMPORTance of the Nucleus during Flavivirus Replication

**DOI:** 10.3390/v9010014

**Published:** 2017-01-19

**Authors:** Adam J. Lopez-Denman, Jason M. Mackenzie

**Affiliations:** 1 Department of Microbiology & Immunology, Peter Doherty Institute for Infection and Immunity, University of Melbourne, Victoria 3010, Australia; adam.lopez@unimelb.edu.au; 2 Department of Physiology, Anatomy & Microbiology, La Trobe University, Melbourne 3086, Australia

**Keywords:** flavivirus, nucleus, transport, karyopherins, nuclear pore complex, nuclear localisation sequence

## Abstract

Flaviviruses are a large group of arboviruses of significant medical concern worldwide. With outbreaks a common occurrence, the need for efficient viral control is required more than ever. It is well understood that flaviviruses modulate the composition and structure of membranes in the cytoplasm that are crucial for efficient replication and evading immune detection. As the flavivirus genome consists of positive sense RNA, replication can occur wholly within the cytoplasm. What is becoming more evident is that some viral proteins also have the ability to translocate to the nucleus, with potential roles in replication and immune system perturbation. In this review, we discuss the current understanding of flavivirus nuclear localisation, and the function it has during flavivirus infection. We also describe—while closely related—the functional differences between similar viral proteins in their nuclear translocation.

## 1. Introduction

**Figure 1:**
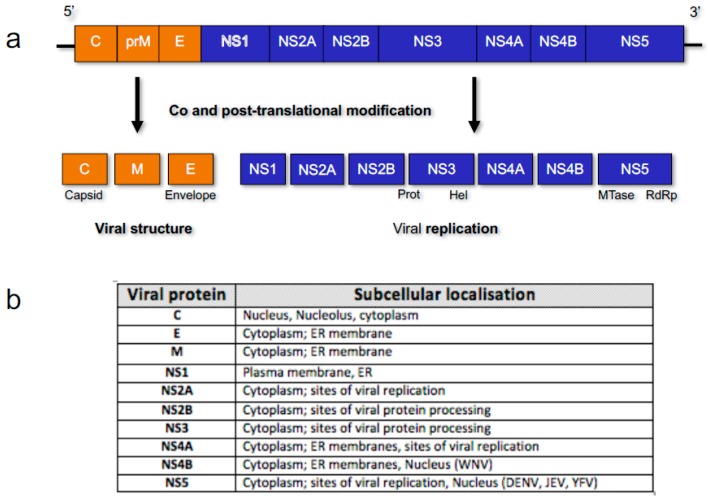
Representation of Flavivirus genome organisation. (**a**) Ten viral genes encode for three structural and seven non-structural (NS) viral proteins that are co- and post-translationally modified into mature viral proteins by host factors and the viral NS2B-3 protease complex [2]; (**b**) Viral protein subcellular localisation is generally cytoplasmic or associated with the endoplasmic reticulum (ER) membrane, due to the localisation of replication, but in specific viruses (listed), proteins such as NS5 can also localise to the nucleus.

Flaviviruses are a large group of diverse arboviruses within the *Flaviviridae* family of viruses that include human pathogens of great medical and social burden worldwide, with billions of US Dollars being spent annually on patient care and vector control. Key members of this group include dengue virus (DENV), Japanese encephalitis virus (JEV), yellow fever virus (YFV), West Nile virus (WNV), and Zika virus (ZIKV). Flaviviruses are seemingly simple viruses, consisting of a positive sense single stranded RNA genome of ~11 kb (with 5′ and 3′ UTRs) that encodes for a single polyprotein that is co- and post-translationally modified into mature proteins. The genes encode for three structural proteins: capsid (C), pre-membrane (prM) and envelope (E); and seven non-structural (NS) proteins, many of which are multifunctional, responsible for viral replication and immune system perturbation [1] (Figure 1).

Flavivirus replication has been extensively studied, and shown to involve the considerable redistribution of host membranes to facilitate efficient replication within the cytoplasm [2,3,4,5]. This membrane redistribution allows for the creation of virally induced structures known as replication complexes (RCs) which act as a replicative niche for the virus in the cytoplasm, and help in shielding from immune detection [5,6,7,8]. The membranous origin of the RC is complex and differs between viruses, but accumulation within the perinuclear region seems common between them [2]. Throughout their replication, Flaviviruses are associated with three distinct structures; the rough endoplasmic reticulum (rER), convoluted membranes/paracrystalline arrays (CM/PC), and vesicle packets (VPs). Due to the nature of the flavivirus positive sense RNA genome, replication can fully occur within the cytoplasm, with no nuclear intermediaries seemingly necessary. Although considered cytoplasmic viruses, in recent years it has come to the attention of many groups that some viral proteins localise to the nucleus during infection or overexpression. When nuclear transport has been inhibited, either by use of mutagenesis or chemical inhibition, there have been many observable effects (with varying levels of significance) to viral replication and modulating host transcription. This has led us, and many groups, to believe that there may be a previously unrealised significant role of viral protein nuclear localisation amongst the flaviviruses.

## 2. Nuclear Trafficking

**Figure 2:**
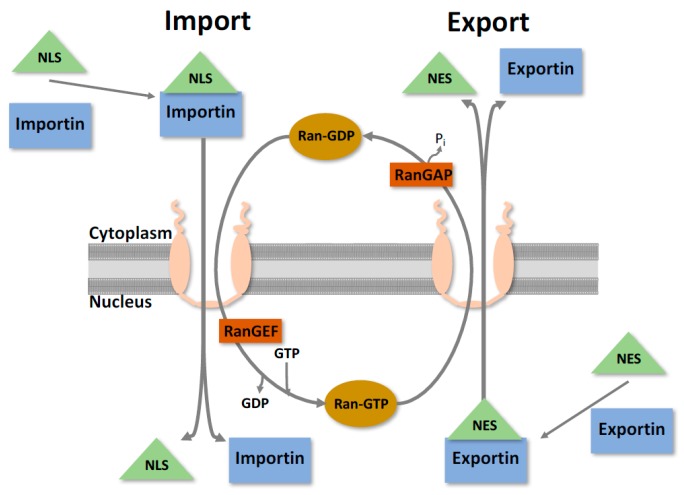
Simplified nucleocytoplasmic protein transport. The nuclear envelope is the physical barrier between the nucleus and the cytoplasm, and is studded with channels known as nuclear pore complexes. For proteins to undergo nucleocytoplasmic transport, they require a nuclear localisation sequence (NLS) or nuclear export sequence (NES), and its corresponding karyopherin. Transport then occurs through the nuclear pore, with directionality determined through interactions with the Ran cycle. When in the cytoplasm, Ran is in a GDP bound state and can facilitate nuclear import. Once in the nucleus, the GDP gets converted to GTP and now can facilitate export [9].

The nuclear envelope is the physical barrier between the nucleoplasm and the cytoplasm, consisting of a double membrane that is studded with large proteinaceous channels known as nuclear pore complexes (NPCs). Both sides of the nuclear membrane exhibit different protein composition; the outer membrane is contiguous with the ER and, like the ER, is associated with ribosomes undergoing active protein synthesis. The inner membrane appears to have more structural functions and is involved with anchoring chromatin and attaching the nuclear lamina [9,10,11]. Bidirectional nuclear translocation is a tightly regulated selective process occurring through the NPC that segregates the contents of the cytoplasm and the nucleoplasm [9] (Figure 2). This is an essential function that facilitates the transport of an enormous range of proteins, ribonucleoproteins, and various macromolecules, while simultaneously preventing non-specific molecules from passing through.

The NPC itself is a very large structure (>120 mDa) made up of ~30 different proteins (known as nucleoporins) in multiple copies, leading to its huge structure of ~400 polypeptides [9]. Free diffusion of small proteins/macromolecules can occur if they are less than 50 kDa in size, anything larger than this needs to undergo active transport to gain access to the nucleus. To facilitate nuclear transport, a specific targeting sequencing and a transport molecular (that recognises the receptor) is required. Nuclear bound trafficking is guided through specific amino acid sequences known as a nuclear localisation sequence (NLS) (typically Arg and Lys), whilst cytoplasmic trafficking is guided by a nuclear export sequence (NES). There are variations of composition of these sequences, with the classical being simply five contiguous basic amino acids (KKKRK), but often they can also be bi-partite sequences containing two clusters of basic amino acids with a spacer ~10 amino acids in between [12,13,14]. Although these two examples have been defined, there is likely a myriad of others that work in similar ways that are currently unknown.

As mentioned, specific molecules are required for nucleocytoplasmic transport to occur, typically by a member of the large superfamily of proteins known as karyopherins (also known as importins, exportins and transportins) [15,16]. Karyopherins mediate transport by binding to an NLS/NES and interact directly with components of the NPC. To further regulate this process, karyopherins can bind to members of the Ras-like GTPase Ran [17]. This helps provide the directionality of nuclear transport by creating a Ran gradient across the NPC. In the cytoplasm, Ran is in a GDP bound state, whilst in the nucleus it is bound to GTP. This is determined by the Ran GTPase activating protein (RanGAP) in the cytoplasm and the Ran guanine nucleotide exchange factor (RanGEF) in the nucleus [17]. In the cytoplasm, a karyopherin binds to its associated NLS/Cargo and forms a protein-complex to facilitate transport to the NPC. As it traverses, it interacts with the RanGTP in the nucleus, releasing the protein and is recycled back into the cytoplasm. Export occurs in an analogous way, with RanGTP affinity being essential within the nucleus, losing binding as it travels through the NPC towards the cytoplasm (as shown in Figure 2).

## 3. Flavivirus Protein Nuclear Localisation

### 3.1. Core/Capsid

The flavivirus structural protein C has been observed to localise to the nucleus across many flavivirus species (DENV, Kunjin virus (KUNV), JEV), and in some cases C nuclear translocation has been shown to enhance viral replication [18,19,20]. C is a small (~12 kb) basic protein with charged N- and C-termini, separated by a hydrophobic region. Although the hydrophobicity remains similar amongst the flaviviruses, the amino acid homology can vary greatly between them. The structure of C has eluded to the RNA binding and membrane interaction functions that have been described during infection [21,22]. During the flavivirus lifecycle, multiple C subunits bind together to form the viral nucleocapsid, encasing the RNA genome, allowing for further pathogenesis. It has also been shown in WNV and tick-borne encephalitis virus (TBEV) that C is permissive to major deletions, with large segments able to be deleted without greatly affecting the ability to encapsulate the viral genome, although they are less infective [23,24,25]. Even so, the underlying role for C nuclear localisation remains poorly understood, however there have been some insights into its function. The introduction of mutations within a putative NLS within JEV C protein were shown to inhibit its nuclear localisation, leading to a reduced amount of infectious virus produced in mammalian cells [20,24,26]. The consequence of this was explained in two potential ways: (i) the introduction of these mutations had an effect on the efficiency of virus particle production; or (ii) a potential role for C in the nucleus not being completed. This is particularly interesting, as it was also observed that similar mutations in TBEV C protein, which has not been shown to go to the nucleus, increased the production of subviral particles, however reduced their infectivity. Interactions with C and the host cell nuclear components are varied between the flaviviruses but include DENV—with Death Domain Associated Protein (DAXX) interaction and Fas-mediated apoptosis [27,28]; JEV-host protein B23, allowing for increased replication [29]; WNV-binding of DDX56, for efficient assembly of infectious particles [30]; and sequestration of HDM2, influencing apoptosis [31].

Even though the nuclear function of flavivirus C is not fully elucidated, groups have mapped predicted NLSs within the protein. There are three proposed NLSs located within DENV at amino acids 6–9, 73–76 and a bipartite sequence found at 85–100 [19,32,33]. Additionally, it has also been identified that in WNV, nuclear import is mediated by importin-alpha/beta, specifically through interactions with amino acids 42–43 and 85–101 [34]. JEV, whilst similar again, showed different NLS functionality compared to the others, with an active NLS being found with amino acids 42 and 43 [20]. It is also speculated that as nuclear transport is often regulated by phosphorylation [35], there may be a phosphorylated C that gains nuclear access and it was shown that specific phosphorylation on residues 83, 99 and 100 influences nuclear localisation (by altering binding efficacy to importin-alpha) [36]. Whilst C protein performs a similar role in viral capsid assembly across the flaviviruses, it is also clear that there are differences in alternate functionality between them.

### 3.2. Non-Structural (NS) Protein 5 (NS5)

Of the NS proteins, NS5 has garnered much interest regarding its nuclear localisation over the past few years. NS5 is a large (~103 kd), highly conserved enzymatic protein with both RNA-Dependent RNA-polymerase (RdRp) and methyltransferase abilities, and has been shown to be intimately involved with viral RNA replication within the RCs. Thus, it is especially interesting that such a large viral protein, particularly one with RdRp activity, may also have a role within the nucleus. During YFV, JEV and DENV infections, there have been demonstrated cases of NS5’s ability to localise to the nucleus [37,38,39]. As NS5 is too large to passively diffuse, the only way it can access the nucleus is through active transport through the NPC. The two enzymatic regions of NS5 are separated by a 37-amino acid linker region (NS5 residue 369–405) in DENV. This region has been thoroughly interrogated, and found to contain two functional NLSs, which have been shown to function through both importin alpha-beta, and importin beta transport [38,40,41,42]. Unsurprisingly, as NS5 is highly conserved amongst the flaviviruses, the NLSs also share this conservation [41]. When nuclear localiation of NS5 was inhibited through mutagenesis of these NLSs, a significant decrease in the amount of virus was observed [41]. Why this is occuring is not fully understood, but may be due to DENV NS5’s ability to modulate IL-8 [43,44], but could also be due to (i) potential structural conformational changes in NS5 that occur from introducing these mutations; or (ii) altering NS5 binding affinity with viral or host proteins during replication [45]. Interestingly, when alanine mutations were introduced to the C terminal of the bipartite NLS, NS5 nuclear translocation was immensely impaired but either did not effect replication, or had only a minimal effect [45]; this further suggests that perhaps nuclear NS5 is not needed, or that only a small portion of cellular NS5 is required within the nucleus. Through the use of the CRM1-inhibitor leptomycin B (LMB), it has also been observed that DENV NS5 can accumulate within the nucleus. This leads to the belief that NS5 also has a NES for nuclear export, and may lead to futher understanding of the function of NS5 upon exit from the nucleus. Rawlinson et al. were able to identify a functional NES on residues 327–343, and confirmed its interaction with CRM1 [43]. While the two aformentioned NLSs have been the most highly characterised, recently a motif within the C-terminal of NS5 has also been shown to be a determinant of subscullular localsiation and RNA binding for DENV 1–4 [46].

Whilst NS5 sub-nuclear localisation has not been fully elucidated, it has recently been shown in DENV that it can localise to the nucleolus in a pH dependent manner [47]. This gives further insight into NS5 nuclear functionality in manipulating cellular responses, and shows that it could be using a multifunctional approach within the nucleus. The majority of this research was performed in DENV-2, however the nuclear localisation of NS5 in all strains of DENV has also been observed, with notable differences in localisation and nuclear accumulation between them [48,49], with serotypes 2 and 3 accummulating more within the nucleus, whereas 1 and 4 do not. Although there are no direct links between NS5 localicalisation and pathogenesis, the exact role that NS5 plays in the nucleus may impact on disease outcome and thus would imply that a targeted inhibition of nuclear transport may only be appropriate for particular strains.

However, it should be noted that a recent paper has revealed that the nuclear localisation of DENV NS5 is neither critical for replication nor the ability of DENV NS5 to impede immune evasion via the degradation of STAT-1 [45]. Via mutation, the investigators showed that mutation of the putative NLS actually impaired the RdRp activity of the mutants’ NS5 species, thus impacting virus replication. Interestingly, the NLS mutants that impaired virus replication could not be rescued in trans, indicating that the links between nuclear NS5 with replication and intracellular survival are not that straight forward and that interactions with nuclear and/or cytoplasmic host factors may be quite complex, influencing the different functions of the flavivirus NS5 protein. These observations are additionally confirmed by the Vasudevan laboratory [46] where they show that the nuclear localisation of some DENV serotype NS5 proteins is mediated via a C-terminal motif and that removal or mutation of this motif in DENV1 results in a completely cytoplasmically localised virus that replicates efficiently. Interestingly, our own reassessment of KUNV NS5 indicates that the protein shuttles very quickly between the nucleus and the cytoplasm, yet does not accummulate within the nucleus as observed for some DENV NS5 proteins (unpublished results). Thus, the exact role for nuclear NS5 in the replication cycle or for immune evasion is still unknown, particuarly if these roles are conserved amongst the different flaviviruses.

In light of these observations, the realisation and significance of nuclear localised NS5 has now been seen to be important in the flavivirus lifecycle, and as such its import pathway has become a target for drug therapy. Through the use of highthroughput screening, thousands of compounds have been able to be analysed for their effectiveness in inhibiting nuclear transport [50]. This has led to the discovery of several compounds that have some effectiveness against blocking DENV infection [51,52]. Ivermectin was one such drug that has been shown to inhibit importin alpha/beta1 nuclear import, the main pathway for NS5 nuclear localisation. When treated with Ivermectin, the inhibition of NS5 and Importin interactions, (therefore stopping NS5 nuclear import) leads to a significant decrease in the amount of DENV present, leading to its potential use as an antivial compound [48,51]. The drawback of chemical inhibitors is that they often have unspecific effects, and could inadvertantly target normal host functions required for cell survival [53]. The benefit of these compounds is that they could have broad antioviral activity against the actions of the flavivirus core, NS5 and potentially NS4B, in the nucleus of infected cells.

### 3.3. NS Proteins NS3 and NS4B and RNA Synthesis

The C and NS5 proteins have received the most attention with respect to nuclear localisation during virus replication and transient expression, however, some reports have also suggested that the flavivirus NS3 and NS4B proteins and RNA synthesis also appear within the nucleus [18,54]. These observations have not been so well defined and are somewhat difficult to interpret, especially the possibility of viral RNA synthesis in the nucleus, as both more sophisticated and specific labelling methods have not consistently detected either viral single-stranded RNA (ssRNA) or double-stranded RNA (dsRNA) within the nucleus [55,56]. In addition, higher resolution confocal microscopy and immunogold labelling of cryopreserved material also reveals a confinement of viral RNA replication in the cytoplasm [8,55,56,57,58,59]. A recent analysis of DENV NS4B has revealed a new role for the protein in mitochondrial localisation but limited protein was observed in the nucleus [60,61]. Overall, the localisation of the NS3, NS4B and viral RNA within the nucleus has not been consistently observed across the flavivirus genus, and thus requires further investigation.

## 4. Sequestration of Host Nuclear Components

While this review is focused on flavivirus protein nuclear trafficking, it is also important to note that proteins encoded within the flavivirus genome also have the ability to sequester cellular nuclear proteins, and inhibit host protein nuclear import. Type I interferon (IFN) is produced in response to viral infections, and plays an important role in innate immunity towards viral infection, with the ability to activate sevaral antiviral pathways [62,63,64]. It is a common theme for flavivirus NS5 to inhibit the IFN mediated Janus kinase/signal transducers and activators of transcription (JAK-STAT) signalling [65,66,67,68], aiding in virus replication. DEAD-box RNA helicases are a large group of proteins, with varied function, that reside in both the nucleus and cytoplasm that have also been shown to be redistributed to sites of flavivirus replication. These have varied functions, but many have been identified as cofactors for replication, or assisting with the antivial response [30,69,70,71,72,73], demonstraing that their sequestration could be essential for flavivirus replication. DDX3 cellular localisaiton has been shown to be both cytoplasmic and nuclear, but is redistributed to sites of JEV replication in the cytoplasm with interactions in binding 5′ and 3′ UTRs, and with NS5 and NS3—proving to be necessary for JEV infection [72]. DDX21 is nuclear localised in uninfected cells, but is redistributed to the cytoplasm during DENV infection, where it has a role in directly inhibiting viruses, and through other intermediaries activating the innate immune response [70,74]. Once in the cytoplasm, DENV NS2B/3 degrades DDX21, aiding viral replication. DDX56 has been shown to redistribute from its normal nucleolar localisation, to sites of WNV replication in the ER [30,69]. This relocalisation interacts with the WNV capsid, with the helicase activity of DDX56 having a role in correct infectious particle assembly [73].

Synthesis of both positive and negative stranded RNA in flaviviruses is a complex process that requires the interaction of viral RNA secondary structures with host nuclear components to facilitate efficient replication (Table 1). These interactions occur at varying stages and have roles in RNA synthesis, genome circularization, and sequestering cytoplasmic proteins [75,76,77]. During DENV infections, a clear distribution of polypyrimidine-tract-binding (PTB) protein from the nucleus to the cytoplasm has been suggested to play a positive role in viral replication, with siRNA knockdown of PTB inhibiting replication, and overexpression stimulating replication [78]. La autoantigen (La) is a ribonucleoprotein found in the nucleus of healthy cells [79]; it has been shown to bind to both the 5′ and 3′ flavivirus UTRs, with the potential role of stabilising RNA loop structure, and/or recruiting NS5 and NS3 [76]. Flaviviruses have also been shown to interact with TIAR/TIA1, another set of proteins that are shuttled from the nucleus to the cytoplasm during cell stress. They have both been shown to interact with WNV 3′ UTR, aiding with viral replication [80]. The additional advantage of WNV sequestration is that TIAR/TIA1 are also associated with the formation of stress granules (SG) and, by inhibiting SG formation, may prevent priming of the innate immune response [80,81].

Although it is yet to be elucidated exactly which viral proteins mediate the sequestration of nuclear proteins within the cytoplasm, it is clear that many flaviviruses have evolved to specifically restrict the intra-nuclear shuttling of many host proteins that aid in the replication of the viral genome but equally restrict host cell immune sensing and communication. It is also yet to be determined if the different flaviviruses selectively bind cellular factors aiding in intracellular replication or globally restrict nuclear trafficking. One tends to favour the former as some flavivirus proteins shuttle in and out of the nucleus and import, rather than export, appears to be a critical requirement for flavivirus replication in cells.

## 5. Future Considerations

From the current literature, we now have some basic insight into the role that the nucleus, and viral protein nuclear localisation has during flavivirus replication. As the flavivirus lifecycle has been mainly observed within the cytoplasm, nuclear transport of flavivirus components has been an underrepresented field of study. Of the flaviviruses, DENV has been the main focus of research, but it is evident that nuclear localisation is occurring amongst other flaviviruses. It is also interesting to note that while the corresponding proteins (i.e., C and NS5) have been observed trafficking into the nucleus across flaviviruses, they may have different functions/roles between related viruses. The targeted inhibition of nuclear transport is also a potential therapeutic target for positive stranded RNA virus infection that has not been robustly interrogated. With the knowledge of nuclear transport being more significant than previously thought, therapies could be developed to target viral nuclear transport mechanisms to combat disease. Finally, most of these studies have been completed in mammalian cells. As a key part of the viral life cycle occurs within arthropod vectors, it would be advantageous to pursue the role, if any, of nuclear localisation within them.

## Figures and Tables

**Table 1:** Sequestered host nuclear factors (adapted from [82]).

Virus	Host Factor	Role in Sequestration
Flavi	La	IRES conformation, translation-replication switching, genome circularization
Flavi	PTB	IRES conformation, eIF4G1 recruitment to IRES, genome circularization
DENV	RNA Helicase A	Genome circularization protein bridge, promote RNA replication
Flavi/WNV	Tia1, TIAR	Promotes + strand RNA synthesis
Flavi/DENV	Tudor-DN/p-100	Promotes RNA synthesis

IRES, internal ribosomal entry site; La, La autoantigen; PTB, polypyrimidine-tract-binding; DENV, dengue virus; WNV, West Nile virus.
